# The potential role of violet-blue light to preventing hospital acquired infections: a systematic review

**DOI:** 10.3389/fpubh.2024.1474295

**Published:** 2024-10-24

**Authors:** Valentina Lucarelli, Davide Amodeo, Isa de Palma, Nicola Nante, Gabriele Cevenini, Gabriele Messina

**Affiliations:** ^1^Department of Molecular and Developmental Medicine, University of Siena, Siena, Italy; ^2^Department of Medical Biotechnology, University of Siena, Siena, Italy

**Keywords:** violet-blue light, disinfection, HAI, surface contamination, prevention

## Abstract

Healthcare-associated infections (HAIs) are a major challenge in modern healthcare, leading to increased mortality, financial burden and negative societal impact. The World Health Organization (WHO) and others have highlighted the alarming rise in HAIs, exacerbated by antimicrobial resistance (AMR), which further complicates treatment. The efficacy of violet-blue light (VBL) technology (approximately 405–420 nm) in inactivating various pathogens and its safety for human exposure have been extensively studied. This study analyses the scientific literature on the use of VBL as a disinfection method in health care settings, with cost and safety implications. It discusses VBL in comparison to other disinfection methods, the implications of its use, and its potential in reducing HAIs due to its ability to be used in occupied environments. While UV technology is more effective at bacterial inactivation, the continuous application of VBL compensates for this difference. UV and VBL technologies have a positive environmental impact, eliminating the need for consumables and reducing waste. Safety concerns are very limited for VBL compared to UV when properly used. The literature highlights that implementing VBL can be a significant step in continuous environmental disinfection in both healthcare and domestic settings. VBL is safe for occupants and offers a feasible, green method for combating environmental contamination and potentially reducing HAIs.

## Introduction

Healthcare-associated infections (HAIs) present a significant challenge in modern healthcare despite available preventive measures and treatments (1). The World Health Organization (WHO) reports that globally, 7 to 15, respectively, in developed and developing countries, out of every 100 patients in acute care hospitals acquire HAIs. Ten per cent of these die, and mortality increases to two to three times when infections are antimicrobial resistant (1).

In the European Union/European Economic Area (EU/EEA), the European Center for Disease Control (ECDC) estimates a prevalence rate of 7.1% (country range 3.1–13.8%) for HAIs in acute care hospitals [8.0% (CI 95%: 6.6–9.6%) prevalence adjusted for the results of national validation studies], translating to an estimate of about 93.305 HAI (CI 95%: 76.427–111.899) patients on any given day and an annual total of 4.3 million (CI 95%: 3.1–5.8%) HAI patients (2). The United States similarly experiences a high burden, with the Centers for Disease Control (CDC) estimating 1.7 million HAIs annually, leading to approximately 99,000 deaths (3).

In intensive care units alone, it is estimated that 8,650 thousands of deaths are attributable to HAI pneumonia and 3.43 million additional days of hospitalization in intensive care units (4).

Economically, HAIs impose a significant burden, costing the U.S. healthcare system $28–45 billion annually and approximately €7 billion per year in Europe (5, 6). These costs include direct medical expenses, at least $28.4 billion and $12.4 external costs from lost productivity and premature deaths (3).

Considering these data preventive strategies and surveillance efforts to mitigate HAIs is becoming and essential priority for the patient safeties and healthcare expenditures.

Adding to this context is the growing problem of antimicrobial resistance (AMR), which threatens the prevention and treatment efficacy of a wide range of infections caused by polychemically resistant bacteria. The emergence of new resistance mechanisms and their global spread threatens the effectiveness of the therapies we must treat infectious diseases. According to ECDC data, 70.9% of cases of infections associated with antibiotic-resistant bacteria were HAIs (7–12).

According to the Organization for Economic Cooperation and Development (OECD) deaths caused by antimicrobial resistant organisms about 79,000 per year. The total annual cost of antimicrobial resistance in the 34 OECD and EU/EEA countries is about US$58 *per capita*, at purchasing power parity (PPP). About one-third of these costs (nearly US$26 PPP *per capita*) are due to increased health care spending and the rest to reduced labor productivity (nearly US$33 PPP *per capita*). Healthcare-acquired resistant infections are responsible for 60% of all deaths and account for about one-third of all resistant infections (13).

The European Parliament says that to maximize the prevention of HAIs and antibiotic resistance, it is essential, among other actions to be put in place, to develop Europe-wide research in HAI prevention and control, including studies on the cost-effectiveness of prevention and control measures (14).

Several studies have been done in recent years to find technologies that effectively reduce the spread of human pathogenic microorganisms to contain the incidence of infections, particularly those acquired in hospital settings (15–21). The most important limitations of the technologies studied, such as ultraviolet (UV) radiation or disinfection methods using chemical detergents, applied to the environmental disinfection, lie in the effects they have on human health when directly exposed and the impossibility of applying them in occupied environments (22–24). However, room contamination occurs in the presence of people and during routine activities (25, 26). Therefore, it is evident how an optimal solution would be to provide constant, effective, and safe room disinfection even in the presence of patients and healthcare workers.

In this context, therefore, the hypothesis was developed, later proven effective and safe over the years, of using violet-blue light (VBL) with a wavelength around 405–420 nm as the disinfection technology (27–30). Violet-blue light near the visible spectrum is not affected by this limitation because its use at controlled doses is safe for humans (31). In addition, advances in LED technology make their adoption attractive because they are economically sustainable and energy efficient (32–34).

The results obtained justify the great interest directed toward this wavelength, as it has also been shown to have a broad spectrum of activity against polychemoresistant bacteria and microbes prevalent in hospital settings (35–37).

The aim of this systematic review is: (i) to analyze the scientific literature compiled on the application of technology with VBL as a disinfection method in health care settings and (ii) to explore the safety of its use, the advantages and disadvantages secondary to its application in hospitals.

## Materials and methods

A literature search was conducted to investigate the potential benefits of the use of VBL in health care. PubMed, Scopus and Google Scholar search engines were used for this purpose. The research of literature occurred at the end of 2023, and it was updated on April 30, 2024. “The Preferred Reporting Items for Systematic reviews and Meta-Analyses (PRISMA) statement” was used to draft the systematic review (38). The keywords used were “violet-blue light,” “VBL,” “405 nm,” “415 nm,” “422 nm,” “photodynamic inactivation,” “disinfection,” “hospital-acquired infection,” “environment,” “hospital,” “cost” and “safety.”

No time or geographic limits were used in the search, and all studies using the keywords were considered regardless of country and year. The keywords were used as follows:

the words “violet-blue light,” “VBL,” “405 nm,” “415 nm,” “422 nm,” “photodynamic inactivation” were each crossed with the word “disinfection”; then the words “violet-blue light,” “VBL,” “405 nm,” “415 nm,” “422 nm,” “photodynamic inactivation” were each crossed with the words “hospital-acquired infection,” “environment,” “hospital”; then the words “violet-blue light,” “VBL,” “405 nm,” “415 nm,” “422 nm,” “photodynamic inactivation” were each crossed with the words “hospital-acquired infection,” “environment,” “hospital,” and each cross was associated with the word “disinfection”; then the words “violet-blue light,” “VBL,” “405 nm,” “415 nm,” “422 nm,” “photodynamic inactivation” were each crossed with the words “disinfection,” “hospital-acquired infection,” “environment,” “hospital” and each cross was associated with the word “cost”; finally, the words “violet-blue light,” “VBL,” “405 nm,” “415 nm,” “422 nm,” “photodynamic inactivation” were each crossed with the words “disinfection,” “hospital-acquired infection,” “environment,” “hospital” and each cross was associated with the word “safety.”

Inclusion criteria were satisfied if the study aimed to analyze the use of violet-blue light in health care settings and whether it could be used in terms of efficacy, safety, or cost. Efficacy was considered as the reduction of bacterial count in the surfaces or air of a healthcare setting or the reduction of HAIs in environments where the technology had been applied.

To further complete the description and perspectives on the use of VBL, scientific literature was then used to discuss the human health risks, evaluated the advantages and disadvantages of using VBL (including comparison with other disinfection techniques), the effect on eukaryotic cells exposed to VBL in human-occupied environments, the current legislation on safety limits, the damage to synthetic and non-synthetic polymers to assess their impact on the degradation of materials in health care environments, the doses required for effective microbial inactivation, the environmental color impact of VBL and the evaluation to the impossibility or non-utility (in terms of efficacy, risk to human health, and cost–benefit ratio) of blue violet light in health care.

No automation tools were used in the process, and three reviewers independently examined each record and report retrieved.

All abstracts of studies identified using keywords were read, and of these, the full text was read of all those that were relevant to the aim of the systematic review.

Of these, all those that dealt with the treatment of infections with violet-blue light and all those that did not involve an application of the technology in health care were discarded. All selected articles that studied disinfection of hospital environments with violet-blue light also included the use of traditional hygiene techniques with common chemical detergents. Articles that included the use of exogenous photosensitizers were discarded.

To reduce the risk of bias, a detailed protocol was predefined to outline the objective of the systematic review, inclusion and exclusion criteria, search strategies, and analysis of results. An independent assessment was conducted by the three reviewers of the scientific literature. All reviewers conducted an exhaustive and thorough search using the same standardized criteria, after which the results obtained by each were compared.

The results obtained were summarized comprehensively. No data were selectively reported to support the research hypothesis.

The studies were synthesized into “results” section in three subsections to distinguish the objective of the systematic review, the use of VBL in health-care settings, from the two macro-topics fundamental to interpreting its applicability, which are human safety and comparison with other disinfection techniques.

## Results

**Figure 1:**
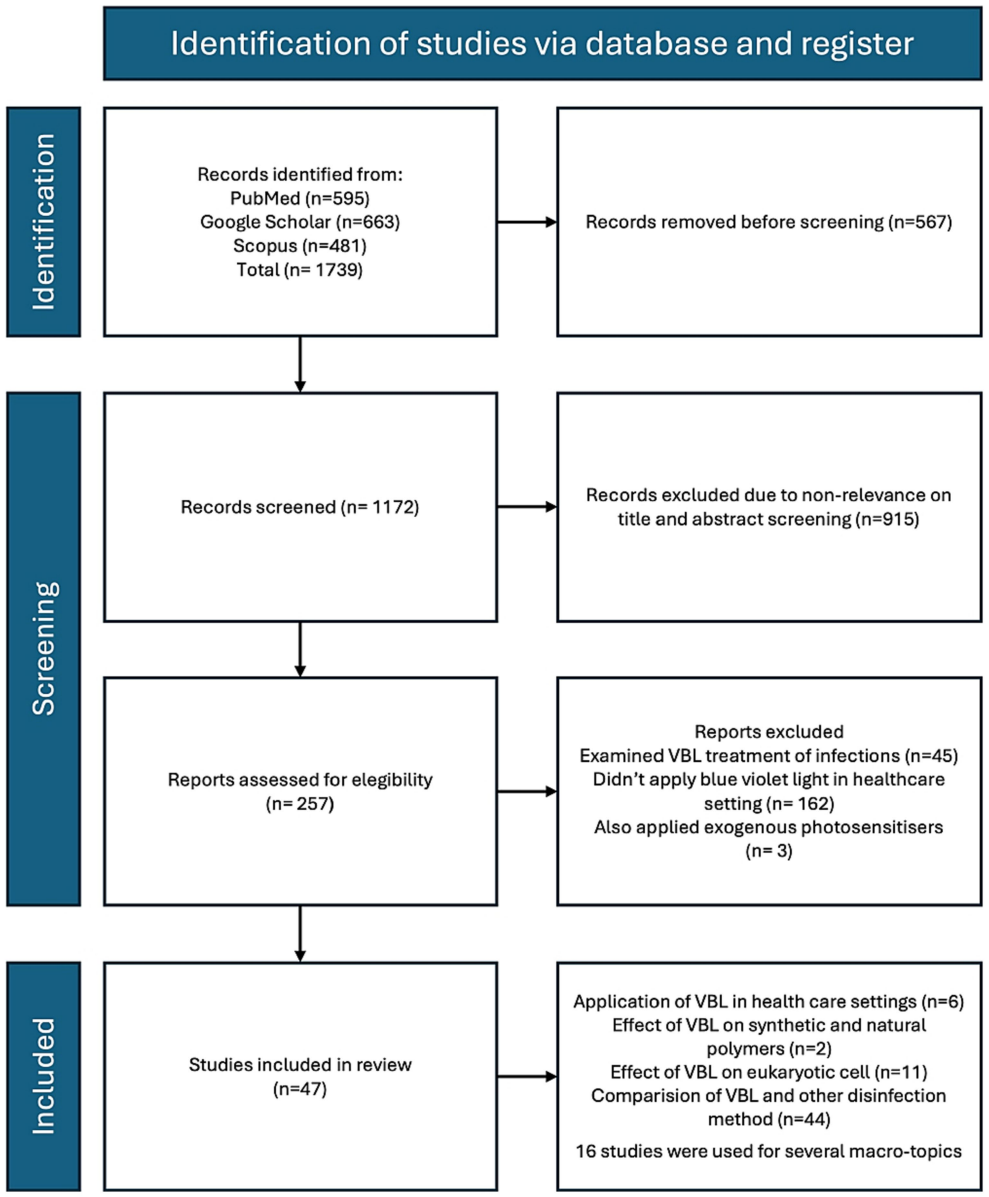
Selection process of studies included in the review.

A total of 1,739 records were identified according to the research criteria. 257 articles were identified as eligibility at the screening but only 47 resulted included in the review. Figure 1 reports the identification of the studies and the selection criteria.

Six articles were identified that investigated the application of violet-blue light in health care settings for preventive purposes (30, 39–43). Of these, only one evaluated the reduction in HAIs following its application and considered the costs associated with HAI reduction. Two articles evaluating the impact of violet-blue light technology on health care materials (44, 45) and 11 studies on eukaryotic cell damage at the doses required for inactivation of health care-related microorganisms were identified (45–56). The latter two aspects investigated were useful in our investigation to go into potential damage to materials and health risks to workers because of continuous exposure.

The subsection “Application of VBL in health-care settings” tabulates and summarizes distinctly the 5 studies that investigated the review objective, the only ones in the literature that met all the inclusion criteria according to the identified keywords. The subsection “effect of VBL on eukaryotic cells” reports all the identified studies that evaluated the effect of VBL on eukaryotic cells. Finally, the third subsection “comparison of VBL and other disinfection methods” reports what are advantages and disadvantages of using VBL compared to other existing techniques (efficacy, dose needed, damage to materials, of interference with health care activities, health risks, cost).

### Application of VBL in health care-settings

A study by Maclean et al. (39) aimed to evaluate the bacterial inactivation efficacy of a “high-intensity narrow-spectrum ambient light decontamination system” (HINS-light EDS) in an isolation room for the treatment of burn patients. Two 405 nm sources were installed on the ceiling of the rooms, and the rate of bacterial inactivation was evaluated in occupied and unoccupied rooms and with the system on and off. Sampling was conducted on environmental surfaces under four different scenarios: unoccupied room, occupied room with EDS off, occupied room with EDS on and constant illumination, and occupied room with EDS on/off, i.e., with intermittent illumination. In all cases of EDS use, a significant reduction in bacterial count was shown compared to disinfection with conventional systems alone. When the room was unoccupied, a 90% reduction in bacterial levels was observed. In the room occupied by a patient with MRSA infection, reductions between 56 and 86% were obtained with continuous lighting. With intermittent EDS activation, bacterial counts were reduced to levels of 62%. In the post-treatment period, the percentages remained low when the environment was unoccupied, while they returned to pre-treatment levels when the environment was occupied, further confirming the usefulness of the technology in constant decontamination of the environment. In this case, 405 nm light was used in conjunction with manual room and surface sanitization, showing that the additive effect of the two methods is greater than traditional decontamination alone.

Bache et al. (40) evaluated the application of a high-intensity narrow-spectrum environmental decontamination system (HINS-light EDS) in inpatient and outpatient units. The ceiling source system was applied in a hospital isolation room occupied by one burn patient and in an outpatient room of the burn unit occupied by 7–12 patients. More than 1,000 samples were taken from the most frequently touched surfaces. A comparison was then made between bacterial contamination levels with and without the use of 405 nm light.

In the isolation rooms, 40 spots (50 spots for patients B and C) were sampled, and 3 consecutive studies (patients A, B, C) were conducted. For each study, samples were collected before the EDS HINS light was used (pre-HINS), after the EDS HINS light had been turned on for 2 days (during-HINS), and after the EDS HINS light had been turned off for another 2 days (post-HINS). In the first study, samples were collected at 8:00 a.m. and immediately after the EDS HINS light was turned on and remained on for the next 2 days during the 14 h light. The other samples were then collected again at 8:00 a.m. after the 2 days of use and then 2 days after being turned off. The study was then repeated by shifting the sampling time to 3:00 pm and 8:00 pm. The study was then repeated with another patient with sampling at 8:00 am and then with a third patient with sampling at 8:00 am for a total of 5 studies in the isolation rooms.

For the samples taken at 8:00 a.m., a significant reduction in environmental contamination levels between 27 and 75% was demonstrated: patient A had a mean reduction in CFU after ESD use of 88.9 (5.7, 183.5; 95% CI) with a percentage reduction of 43%; Patient B had a mean reduction in CFU after ESD use of 16.9 (6.4, 27.4; 95% CI) with a percentage reduction of 75%; Patient C had a mean reduction in CFU after ESD use of 6.8 (−18.6, 32.1; 95% CI) with a percentage reduction of 27%. A lower reduction was observed in samples taken at 3:00 p.m. and 8:00 p.m., probably related to the different activity levels in the room and thus a higher degree of environmental decontamination.

In the study conducted in the outpatient setting, 50 samples were collected at the beginning and end of outpatient activity, with and without the EDS HINS light on for 8 h. The use of 405 nm light resulted in a smaller increase in bacterial counts for samples collected at the end of the outpatient clinic. The ambulatory in which 405 nm light was not used showed an increase in bacterial CFU count from 8.1 CFU/plate to 22.2 CFU/plate with an average increase in bacterial count of 14.1 CFU/plate during the ambulatory. When 405 nm light was used, the increase was from 6.5 CFU/plate to 12.0 CFU/plate with an average increase of 5.5 CFU/plate. The amount of additional room contamination released during the outpatient clinic was reduced by an average of 8.6 CFU/plate (1.4, 15.8; 95% CI). This is equivalent to a significant efficacy of 61% (*p* = 0.02). Complete bacterial abatement was not achieved, but the efficacy of a 405 nm source as an additional tool to chlorine and disinfectant wipes disinfection technologies used prior to exposure to reduce bacterial contamination in health care facilities was still demonstrated.

Another study conducted by Bache et al. (41) was aimed at evaluating the relationship between irradiation and the rate of bacterial inactivation in a hospital burn unit. The study did not demonstrate a significant correlation between irradiation levels and bacterial count reduction, while it showed that increased exposure time was strongly correlated with reduced contamination. The effectiveness of 405 nm light in reducing bacterial counts was also demonstrated with a 22 to 86% decrease in the average number of bacterial colonies. Furthermore, when the light was turned off, an increase between 78 and 309% was shown.

Maclean et al. (42) conducted a study where the same protocol was replicated three times to evaluate the effectiveness of an EDS HINS-light system in environmental disinfection of an intensive care unit and to test how the location of the light source affects the rate of bacterial inactivation. Two units were installed on the ceiling above the areas of highest activity. The irradiance levels were within safe limits according to international guidelines (23), and the systems operated from 07:30 a.m. to 10 p.m. in the presence of patients and staff. The studies were conducted on two patients. In study 1, the patient had been hospitalized for 9 days when the EDS HINS light was turned on. Pre-HINS sampling, before the system was turned on, and HINS sampling, during operation 5 days after the system was turned on, were conducted. A mean reduction after the use of EDS of 19.4 CFU/plate (11.4, 27.4; 95% CI) was demonstrated, which is equivalent to a 66.8% (*p* = 0.0001) reduction in staphylococcal counts. In study 2, the patient was admitted 12 h before the experiment and the pre-HINS count was relatively low. However, a 38% reduction was demonstrated the morning after the system was turned on. The post-HINS count in study 2, with samples taken 24 h after the 405 nm light was turned off, showed a 357% growth in staphylococci.

A third study was conducted to evaluate the effect of source location. It was seen that surfaces exposed to direct light at 405 nm showed a 63% reduction in bacterial counts, with 94% increase 24 h after the light was turned off. Sample counts taken on surfaces exposed to indirect light showed a 48% reduction in bacterial counts and, after the light was turned off, a 71% increase.

A 2016 study by Murrell et al. (43) investigated the impact of a continuous environmental disinfection system (CED) with visible light (Indigo-Clean, Kenall, Kenosha, WI) on surface environmental contamination and the prevention of surgical site infections in an operating room. The CED system analyzed was complementary to the other disinfection methods (combined cleaner and disinfectant used together with a microfiber cloth) and consisted of an LED system with a spectral profile between 405 nm and 410 nm integrated into ambient lighting. The object of the study was three operating rooms dedicated to orthopedic surgery, two of which (OR1 and OR2) shared the same ventilation, heating and air conditioning system while the third did not (OR3). The visible light CED system was installed in OR2 operating room. OR3 was included in the analysis of Surgical Site Infection (SSI) data but not for environmental sampling. The two adjoining rooms (OR1 and OR2) were the sampled rooms. In one of the two rooms, the CED system and surface sampling were done before and after it turned on surfaces frequently touched by operators. Surface samples were collected 5 times in period 1 (pre-CED) and 8 times in period 2 (post-CED) in both operating rooms. Fifty samples were collected each time. Samples in both ORs were collected between 5 and 6 a.m., before the first entry into the operatory room and after the room had been cleaned the previous evening.

In OR2 (room where CED was installed), there was a mean reduction of 81% (*p* = 0.017) in CFU count and a median bacterial reduction of 85% (*p* = 0.002) between periods 1 (before installation of the CED system in OR2) and 2 (after installation of the CED system in OR2). In the contiguous room OR1, which did not have its own CED system but had a ventilation system shared with OR2, a statistically significant mean and median reduction of 49% was observed (*p* = 0.015; *p* = 0.006). The second result of interest was the effect of the CED system on SSI rates.

SSIs in both ORs (OR1 and OR2) and the distant OR (OR3) were monitored for 1 year before and after installation of the visible light CED system. Data on SSI cases from each operating room were collected for 1 year, without CED system use, and then in the following year, with CED system use. In the first period, without CED, 2,201 surgical cases were performed, while in period 2 there were 2,317. It was observed that the SSI rate in the second room decreased from 1.4% (period 1) to 0.4% (period 2). Statistical analysis showed that only 3 of the 12 expected infections occurred, suggesting that 9 potential infections were prevented by the violet-blue light system. A reduction in the SSI rate in contiguous room 1 from 1.2 to 0.3% was also demonstrated, which however, was not found to be statistically significant.

A study by Amadeo et al. (30) in 2023 demonstrated the potential role of 405 nm violet-blue light LEDs in microbial inactivation on high-contact surfaces in a hospital infection control laboratory. Two experiments were conducted in the study. In the first one, high-contact surfaces were sampled before and after 7 days of exposure to the violet-blue light. In the second, the effect of violet-blue light on MRSA-contaminated surfaces was studied. The study was conducted in a 14 m^2^ laboratory, and the lamps used consisted of a 60×60 ceiling light fixture equipped with 12 Nichia NVSW219FT white LEDs (Nichia, Anan, Japan) and 69 Luminus SST-10-UV violet-blue light LEDs (Luminus, Sunnyvale, CA, United States) with a wavelength at 405 n and a power of 1.3 watts each.

In the first experiment, the duration of exposure to violet-blue light in the laboratory was 7 consecutive days. Each day the lamps were turned on at 2:00 pm and turned off at 8:00 am the next day. During the exposure, the activities were finished, and the scenario was static and closed.

The second experiment, conducted on MRSA colonies, was performed on an additional day with the same exposure time as the previous days (18 h). The energy consumption per ceiling light for the 18 h was 1,600 W.

In the first experiment, the high-contact surfaces were sampled at time 0, before exposure, and then on days 1-3-5-7, after irradiation with the 405 nm LED ceiling lights. The heights of the sampled spots ranged from 90 cm to 130 cm. Sampling was done before the start of activities except on day 3 when it was done at 2 pm. Exposure to violet-blue light reduced the number of CFUs in all sampled areas after 24 h. A significant reduction (*p* < 0.05) was observed for desk and keyboard sampling with a reduction at day 1 compared to day 0 of 78.13 and 86.38%, respectively, and at day 7 of 87.50 and 89.96%, respectively. The incubator handle had a low total microbial load already at the beginning of the experiment and no growth was observed after exposure on days 1, 5 and 7. On day 3, the only day when sampling was performed after activities, microbial growth was detected (1 of 3 plates).

In the second experiment, the bactericidal effect of violet-blue light on bacterial suspensions of MRSA distributed on nonporous plastic and steel surfaces was evaluated. Aliquots of 100 μL of a bacterial suspension (2×10^4^/mL) of MRSA were distributed on selected surfaces with a surface area of 55 mm diameter.

The surfaces were exposed to violet-blue light for 18 h. The positive control were parallel areas adjacent to the selected spots on which the same suspension had been distributed and then covered with 3 layers of aluminum to avoid irradiation. On all contaminated surfaces the level of irradiation was significant (*p* < 0.05). The percentage reduction of MRSA microbial load on the exposed surfaces compared with controls was 91.8% on the keyboard, 94.0% on the worktable, and 98.3% on the laboratory sink.

### Effects of VBL on synthetic and natural polymers

Healthcare materials and instrumentation are affected by interaction with disinfection techniques that can change the structural composition and thus the technical properties of the instrument (44). Alterations in the polymeric structures also affect the macroscopic level, resulting in early degradation of the instrument and degradation of the technical properties that are essential for optimal functioning and necessary to ensure safety standards. To assess how and how much VBL impacts this aspect, studies have been conducted on the main structural materials of healthcare instrumentation.

A study conducted by Irving et al. (44) compared the effects of UVC radiation and violet-blue light on flexible endoscopes with the objective of assessing the level of degradation induced by the different wavelengths. The specimens were exposed for 400 h to a 55 W germicidal UV fluorescent tube peaking at 254 nm and a series of 9 LEDs peaking at 405 nm at an irradiance of 2.6 mW/cm^2^. The material investigated was based on poly (methyl methacrylate) (PMMA). It was shown how the use of UVC on endoscopes led to degradation of the material, resulting in cracking on the material and increased clinical risk associated with the surface irregularities generated. In contrast, light at 405 nm did not lead to alteration of the materials. Photodegradation of the polymers was also analyzed and a change was seen in the Fourier Transform Infrared Spectroscopy (FTIR spectra), this was interpreted as a secondary effect to the cleavage of partial and complete side chains. Subsequently, an increase in cracking and surface roughness visible by light microscopy after exposure to UVC was described, and these aspects were considered as precursors of cracking and blistering described in the warnings regarding the use of UVC in the storage of flexible endoscopes. In addition, these structural changes lead to hydrophobicity of the instrument that goes to alter its effectiveness of use. All these changes were not observed in the material exposed to VBL. In addition to the influence this has on the life of the endoscope and the need to replace it a key aspect is that associated with infectious and clinical risk.

A subsequent study (45) evaluated the effect of violet-blue light on polymethyl methacrylate (PMMA) resin, used as the base material for prostheses, which is at high risk of contamination and a frequent cause of prosthetic stomatitis.

For the CFU test, 80 polymethylmethacrylate (PMMA) denture resin discs contaminated with *Candida albicans* and Candida glabrata biofilms were prepared. The samples were irradiated with LED sources emitting wavelengths at 405 nm (Osada Electric Co. Ltd., Tokyo, Japan). A spectrophotometer was used to confirm the wavelength of the radiation emitted by the LED.

Prosthetic resin discs with *Candida albicans* and *Candida glabrata* biofilms were irradiated with 405 nm light at various irradiation times: 0 min (control), 5 min (47.4 J/cm^2^), 10 min (94.8 J/cm^2^), 20 min (189.6 J/cm^2^) and 30 min (284.4 J/cm^2^). Then resin discs irradiated for 0 min (control) and 20 min (189.6 J/cm^2^) were sectioned and further examined by fluorescence microscopy and scanning electron microscopy (SEM). Ten resin discs were analyzed for each group (control and 189.6 J/cm2). A reduction in CFU count was observed for both *C. albicans* and *C. glabrata* species, with reduction in biofilms as exposure times increased.

For post-irradiation characterization of the materials, 15 parallelepiped specimens (64 × 10 × 3.3 mm^3^) of PMMA denture base resin were prepared. The specimens were polished with 1,000-grit sandpaper under running water using a polishing device. The specimens were irradiated on one side only with light at 405 nm at constant power of 280 mW/cm^2^. Irradiation times were: 0 h (control, *n* = 5), 84 h (84,672 J/cm^2^; *n* = 5) and 168 h (169,344 J/cm^2^; *n* = 5).

Irradiation of a prosthesis for 84 h and 168 h is equivalent to a daily exposure of 30 min for 6 months and 1 year, respectively. After irradiation, the samples were stored in the dark and room temperature for 1 month. Then all samples were stored in deionized water at 37° for 50 h.

Using a three-point bending test, in accordance with the World Organization for Standardization (ISO) 1,567, flexural strength (FS) and flexural modulus (FM) were calculated. In addition, the surface roughness (Ra)of each specimen was determined before testing. To evaluate the effect of 405 nm light on PMMA, FS, FM and Ra pre-irradiation and post-irradiation of each sample were studied. FS was significantly higher in both irradiated groups (> 105.4 MPa) than in the control group (*p* < 0.05), with no significant differences between groups. The FM in all irradiation groups ranged from 2.80 to 2.93, with no significant differences between groups (*p* > 0.05). The Ra of the samples in all groups ranged from 0.19 to 0.20 μm, with no significant differences between groups (*p* > 0.05). For heat-curable acrylic resin. The minimum FS must be >65GPa and the minimum FM >2GPa. In the study, the values were > 105.4 GPa and > 2.8 GPa, respectively, and thus conditional to ISO 1567 even after 168 h of exposure. It should be mentioned, however, that although cleavage of the main chain of PMMA occurs for wavelength values <320 nm, the FS was significantly higher in this study than in the control group. The cause could be possible polymerization of PMMA due to irradiation. The authors themselves state the need for further studies to clarify this phenomenon. In contrast, there were no significant changes in FM and Ra. Surface degradation increases Ra, indicating that irradiation does not induce any deterioration of the resin surface.

### Effects of VBL on eukaryotic cells

The most interesting aspect of the potential use of violet-blue light as a hospital disinfection technique is associated with the possibility of using it in occupied environments (27). Like any prevention and treatment technique, the risks should not outweigh the benefits, and even minimal risk to people would imply that this technology could not be applied in the presence of health care workers and patients (31). However, this is precisely the key of this technology because, as we have said, at the same dose, UV radiation is much more effective than blue violet light, and the advantage in using the latter lies in the possibility of using it at the time of maximum contamination, that is, in the presence of humans (57) (Table 1).

**Table 1:** Results of violet-blue light effects on eukaryotic cells.

Eukaryotic cells	Source	Wavelength (nm)	UV dose	Intensity	Log reduction CFU	Effects on eukaryotic cells	Source
Fibroblast-populated collagen reticulocytes (FPCLs)	HINS light	405 nm		0.8–1.8 mW/cm^2^ × 1 h		No significant inhibitory effect on FPCL contraction. No significant decrease in cell number. Expression of a-SMA protein was unaffected.	(47)
Fibroblast-populated collagen reticulocytes (FPCLs)	HINS light	405 nm	54 J/cm^2^	15 mW/cm^2^ × 1 h		Contraction of FPCLs halts within 24 h of exposure, and the cells do not appear to recover contractile function significantly within 140 h of the exposure. ∼80% reduction in cell number and no significant recovery within 120 h. Expression of the *α*-SMA protein is also inhibited by this higher intensity of HINS light	(47)
Fibroblast-populated collagen reticulocytes (FPCLs)	HINS light	405 nm	(13–54 J/cm^2^)	5 mW/cm^2^ (3.6–15 mW/cm2) × 1 h	3 log *S. epidermidis*	The maximum intensity at which HINS light did not cause an inhibitory effect was established as 5 mW/cm^2^.	(47)
Osteoblast	HINS light	405 nm	54 J/cm^2^	15 mW/cm^2^ × 1 h		Cell morphology, alkaline phosphatase activity, collagen synthesis, and osteocalcin expression were significantly reduced.	(48)
Osteoblast	HINS light	405 nm		5 mW/cm^2^ × 1 h-2 h	98.1% of *S. aureus* 83.2% *S. epidermis*	No effect on cell morphology, activity of alkaline phosphatase, synthesis of collagen or osteocalcin expression, demonstrating that under these conditions this dose is the maximum safe exposure for osteoblasts	(48)
Rat osteoblasts	HINS light	405 nm	4.5 J/cm^2^		98% *A. baumannii*	There were no significant effects on cell viability, function (alkaline phosphatase activity), and proliferation rate of osteoblasts.	(49)
Rat osteoblasts	HINS light	405 nm	18 J/cm^2^	5 mW/cm^2^ × 1 h	100% *S. aureus, S. epidermidis P. aeruginosa, A. baumannii, E. coli, K. pneumoniae*	There were no significant effects on cell viability, function (alkaline phosphatase activity), and proliferation rate of osteoblasts.	(49)
Rat osteoblasts	HINS light	405 nm	36 J/cm^2^	10 mW/cm^2^ × 1 h 5 mW/cm^2^ × 2 h 3.3 mW/cm^2^ × 3 h 2.5 mW/cm^2^ × 4 h	100% *S. aureus, S. epidermidis P. aeruginosa, A. baumannii, E. coli, K. pneumoniae*	There were no significant effects on cell viability, function (alkaline phosphatase activity), and proliferation rate of osteoblasts.	(49)
Rat osteoblasts	HINS light	405 nm	54 J/cm^2^			There was a reduction in cell viability, functionality (alkaline phosphatase activity) and proliferation rate of osteoblasts.	(49)
Skin	Photodynamic therapy (PDT) lamp	420 nm (380–480 nm)	20 J/cm^2^ (cumulative dose 100 J/cm^2^)			No inflammatory cells or scald cells. No change in p53 or MMP-1 expression. No signs of elastosis. There was a significant increase in the perinuclear vacuolisation of keratinocytes and a histological increase in Melan-A-positive cells, expression of clinical hyperpigmentation. No deoxyribonucleic acid damage, no premature photonic aging. There was transient melanogenesis and perinuclear vacuolisation without subsequent apoptosis.	(50)
Keratinocytes	LED	415 nm	70.2 J/cm^2^		5.42 log *C. albicans*	Keratinocytes had a loss of viability of 0.11 log.	(58)
Human vaginal cells	LED	405 nm	108 J/cm^2^		6 log *N_._ gonorrhoeae*	The cytotoxicity study on normal human vaginal epithelial cells (VK2/E6E7) showed no statistically significant loss of cell viability. VBL-induced DNA damage in VK2/E6E7 cells was not induced up to doses of 216 J/cm2.	(51)
Human vaginal cells	LED	405–470 nm	108 J/cm^2^			Selective inactivation of bacterial cells over vaginal cells was demonstrated at doses of 108 J/cm2 with a wavelength of 405 nm.	(52)
Murine brain	LED	405 nm	36/45/54 J/cm^2^		Cultures with 106 and 107 cfu/mL of *E. cloacae* were exposed to 54, 45 and 36 J/cm2 light and counted at 24 h after implantation, revealing a 104 orders of magnitude attenuation of bacterial growth at the highest dose. According to the two-factor ANOVA, bacterial survival (N: N0 ratio) was significantly (*p* < 0.05) affected by exposure dose.	There are no post irradiation behavioral changes or disturbances in mice. Postmortem evaluation of the histologic preparation also showed no abnormalities in the production of glial caspase 3 and fibrillary acid protein nor production of markers of apoptosis and necrosis.	(53)
Plasma	LED	405 nm	360 J/cm^2^		**Low bacterial cell densities (10**^**1**^**–10**^**3**^ **CFU mL**^**−1**^**):** 2.6 log *S. aureus, S. epidermidis, B. cereus, E. coli e P. aeruginosa* 2.55 log *A. baumannii* 2.35 log *Y. enterocolitica* 2.41 *K. pneumoniae* **Medium bacterial cell densities (10**^**4**^**–10**^**6**^ **CFU mL**^**−1**^**):** >4.5 log *Y. enterocolitica, K. pneumoniae* >5.0 log *S. epidermidis, P. aeruginosa, A. baumannii*	The electrophoretic patterns of plasma exposed to the effective antibacterial dose of 360 J cm − 2 did not demonstrate visually detectable differences between the exposed and nonexposed plasma samples, indicating that the antibacterial effect can be achieved with no obvious damage to the plasma proteins.	(46)
Human platlet	LED	405 nm	288 J/cm^2^	10 mW/cm^2^ × 8 h	>99% *S. aureus*	Recovery of the human PLTs show that treatment with antimicrobial 405 nm light had minimal effect on the recovery of human PLTs in the SCID mice when compared to the control, non-treated human PLTs, with no significant differences detected at any of the tested time points (2–10 h; *p* = >0.05).	(54)
**Human platlet**	**LED**	***405 nm***	***270 J/cm2***	***5 h***		***x vivo treatment of human platelets with antimicrobial 405 nm violet-blue light leads to mitochondrial metabolic reprogramming to survive the treatment and alters a fraction of platelet proteome.***	(55)

The analysis of the impact on eukaryotic cells has therefore been the subject of numerous studies to determine, in terms of safety, whether these wavelengths can be used in occupied environments and especially at what doses and for how long.

Mcdonald et al. (47) studied the effect of high-intensity narrow-spectrum light (HINS light) at 405 nm on fibroblast-populated collagen reticulocytes (FPCLs) to evaluate its potential use in wound disinfection. The effect of HINS light on the contraction of FPCLs was examined, and the effects on cell proliferation, morphological changes, and smooth muscle actin expression *α* (α-SMA) were evaluated. The dose required for CFU inactivation of *Staphylococcus epidermidis* and cell viability at the same dose was evaluated. HINS light was shown to have an intensity-dependent inhibitory effect on fibroblast function. One-hour exposure at low intensities (0.8 and 1.8 mW/cm^2^) did not inhibit FPCL contraction, alter α-SMA expression, or produce cell reduction. In contrast, altered cell viability was observed at higher doses of HINS light. 54 J/cm^2^ administered for one-hour at an intensity of 15 mW/cm^2^ altered fibroblast function. Within 24 h there was cessation of contraction of FPCLs, with no recovery within 140 h of exposure, and an 80% reduction in cell number, with no significant recovery within 120 h. This was also confirmed by microscopic imaging, which showed an alteration in fibroblast morphology after exposure at 15 mW/cm^2^ and no effect at low intensities. The highest intensity at which HINS light did not cause an inhibitory effect on FPCL contraction was at 5 mW/cm^2^ for one-hour. At these doses there was significant inactivation of even the *S. epidermidis* colonies studied.

Subsequently, Mcdonald et al. (48) conducted *in vitro* studies with exposure of cultured osteoblasts to violet-blue light at 405 nm (HINS light) with an intensity of up to 15 mW/cm^2^ for 1 hour (54 J/cm^2^). They saw how up to doses of 5 mW/cm^2^ for 1 h showed no changes in osteoblast cell function and no effects on cell morphology, alkaline phosphatase activity, collagen, or osteocalcin expression synthesis. At doses of 15 mW/cm^2^, however, a reduction in all parameters of cell function was shown. The 2 h exposure at 5 mW/cm^2^ did not affect osteoblast viability. In the same study, violet-blue light was shown to be effective as a bactericide at these doses, inactivating 98.1% of CFUs of *Staphylococcus aureus* and 83.2% of CFUs of *S. epidermidis* both laboratory strains and strains isolated from infected arthroplasties.

A study (49) investigated the effects on rat osteoblasts exposed to increasing doses of violet-blue light at 405 nm. Cell viability, functionality (alkaline phosphatase activity) and proliferation rate of osteoblasts were evaluated. Exposures up to doses of 36 J/cm^2^ had no significant effect on cell viability while at doses of 54 J/cm^2^ this was significantly affected. At the same time, it was shown that at doses of 36 J/cm^2^ there was inactivation of several human pathogenic microorganisms. The study thus further demonstrated how the safety of violet-blue light depends on the irradiation dose and how bacterial and osteoblastic cells have different sensitivity to irradiation with 405 nm light.

A study by Kleinpenning et al. (50) aimed to evaluate the effect of violet-blue light on the skin by analyzing photodamage (p53, vacuolization, sunburn cells), photoaging (elastosis, MMP-1) and possible melanogenesis (Melan-A). No inflammatory cells or sunburn cells were evident at the end of the investigation. No change in p53 or MMP-1 expression was observed. Also, no signs of elastosis were seen. However, a significant increase in perinuclear vacuolization of keratinocytes and a histologic increase in Melan-A-positive cells, an expression of clinical hyperpigmentation, was shown. Thus, it was shown that violet-blue light does not cause deoxyribonucleic acid damage or premature photon-aging, leading only to transient melanogenesis and perinuclear vacuolization without subsequent apoptosis.

Zhang et al. (58) evaluated the susceptibility of human keratinocytes versus *C. albicans* colonies to irradiation with VBL at 415 nm. It was shown that *C. albicans* was approximately 42 times more susceptible than human keratinocytes. At doses of 70.2 J/cm^2^, a CFU inactivation of 5.42 log_10_ was observed while keratinocytes had a loss of viability of 0.11 log_10_. The average inactivation rate coefficients of *C. albicans* and keratinocytes, estimated from the slopes of the inactivation curves, were 0.0795 and 0.0019 J/cm^2^, respectively.

Two studies conducted by Wang et al. evaluated the genotoxicity induced on human vaginal cells by violet-blue light sources at the doses required for inactivation of *Neisseria gonorrhoeae*. In the first study, conducted in January 2019, a 405 nm source was used. At doses up to 108 J/cm^2^, a reduction of more than 6 log_10_ CFUs of *N. gonorrhoeae* was observed, while the cytotoxicity study on normal human vaginal epithelial cells (VK2/E6E7) showed no statistically significant loss of cell viability. VBL-induced DNA damage in VK2/E6E7 cells, assessed with a Comet Assay kit, was not induced up to doses of 216 J/cm^2^ (51). In the next study, the role of wavelengths was evaluated and therefore doses from 405 nm to 470 nm were used. Also in this study, selective inactivation of bacterial cells over vaginal cells was demonstrated at doses of 108 J/cm^2^ with a wavelength of 405 nm. In addition, no absorption peaks were observed in the spectrum around 400 nm for VK2/E6E7 cells in this study (52).

Safety evaluation of an LED device at 405 nm for direct antimicrobial treatment on murine brain showed that at doses of 36, 45, and 54 J/cm^2^ there are no post irradiation behavioral changes or disturbances in mice. Postmortem evaluation of the histologic preparation also showed no abnormalities in the production of glial caspase 3 and fibrillary acid protein nor production of markers of apoptosis and necrosis (53).

Three studies have been conducted on human plasma to evaluate bacterial inactivation efficacy and blood cell sensitivity.

Stewart et al. (46) demonstrated that irradiation with VBL at doses of 360 J/cm^2^ does not produce signs of degradation of plasma proteins, preserving their integrity. Maclean et al. (54), in a study to evaluate the potential use of VBL in the prevention of transfusion-transmitted infections, identified superimposable recovery of platelets treated at doses of 180 J/cm^2^ with untreated platelets. Jana et al. (55) demonstrated that *ex vivo* treatment of human platelets with violet-blue light induces mitochondrial metabolic reprogramming to survive treatment.

### Comparison of VBL and other disinfection methods

Numerous studies have demonstrated the role of environmental, airborne and surface contamination in the transmission of healthcare associated infections (59–68), and subsequently numerous efforts have been made not only to optimize the effectiveness of traditional disinfection methods but also to find alternative or complementary technologies (20).

In terms of efficacy, VBL has been shown to be effective in inactivating bacterial species that are pathogenic to humans and responsible for a high rate of nosocomial infections (69–73). In addition, the dose must be further reduced in the presence of people to ensure safety standards (74). This is an important limitation of violet-blue light as doses at these levels may be ineffective for spore and biofilm inactivation (47, 75). However, since it can be used, at controlled doses, in the presence of people, it could be applied in occupied environments and so at the time of maximum contamination (47). Indeed, it has been shown that optimal cleaning is nevertheless followed by rapid recontamination when the room is occupied (25). Commonly used disinfection methods cannot be applied in occupied rooms, which greatly limits their use and the result (76). Commonly used disinfection methods require constant supervision and trained and experienced operators, whereas VBL is not operator-dependent and therefore disinfection effectiveness is guaranteed regardless of who uses it, regardless of the degree of experience (28, 77).

There are limitations to the use of this technology, those related to safety will be described later in this section. An important aspect to consider is the impact a light source can have in coloring the environment and how much this can interfere with routine activities (27). One potential advantage of LED technology is the ability to mix different color spectra to negate this problem (43). In terms of effectiveness, the limitation of all disinfection technologies using light, whether VBL or UV, is that the treatment does not reach shaded and occluded areas and therefore can only be applied to exposed or reflected surfaces (35). In addition, they cannot be used for macroscopic cleaning of air and surfaces. However, their use allows disinfection of air and water, an aspect not possible with mechanical disinfection (59, 78).

Disinfection with light avoids the use of consumables, disinfectants and detergents that result in the production of waste whose disposal has a huge economic and environmental impact (79). The half-life of the light source is long enough to avoid the need for short-term disposal; therefore, the light source can be reused many times (80).

The reviewed studies do not show damage to natural and synthetic polymers post exposure to violet-blue light, unlike the evident structural alteration secondary to prolonged exposure to UVC light. However, in the study by Tsutsumi-Arai et al., significantly higher values are shown for the flexural strength of the resin post-irradiation. This is likely attributed to the possible polymerization of PMMA due to irradiation (44, 45, 81).

About the safety associated with the use of Light-Emitting Diode (LED) sources as a disinfection technique, the occupational risks associated with the use of LEDs are significantly lower than those associated with the use of common cleaners. We refer to the risk of falling and slipping, the potential risk of poisoning or intoxication, electrocution, and burns. All these risks are associated with the improper use of chemical cleaners, but they can be accidental events that can affect even the most experienced personnel (82).

Studies have shown that bacterial cells, at the same dose, are much more sensitive than eukaryotic cells, providing a high degree of safety (47–50, 52, 58). UV, on the other hand, is associated with high photobiological risk, with photoaging and carcinogenesis, regardless of dose (83, 84).

The use of VBL for disinfection is not without risk. The health risks associated with exposure to optical radiation are mainly related to wavelength, exposure time, and thus radiation dose.

Wavelengths within the visible spectrum can cause adverse health effects, as can if the wavelength goes into the UVA spectrum. At 440 nm, the risk of photoretinitis increases, and at 480 nm, the peak of retinal ganglion cell sensitivity, alterations in physiological responses to light, such as circadian physiology and pupillary constriction, can occur (23, 85).

The peaks of light generated at the frequency of 405 nm are not homogeneous. In fact, when the peak is at 405 nm it is possible to generate tails on the left side of the spectrum that can go into the ultraviolet radiation. This poses a risk because photobiologically hazardous energy is present at this level of the spectrum (35). To ensure safe technology, according to EN62471 standards, one must go to assess and control the amount of this energy (31). By adjusting the light emission spectrum, however, one could go and modulate and adjust this parameter, which is possible with LEDs and not with lamps, optimizing the safety of a violet-blue light system (43, 86).

## Discussion

Despite growing interest in VBL for reducing bacterial contamination in healthcare settings, particularly to contrast HAIs and the rising threat of AMR pathogens, *in vivo* studies remain scarce (30, 39–43). While VBL’s efficacy against human pathogens common in HAIs has been well-demonstrated *in vitro* (70–74), further research is needed to assess its cost-effectiveness in real-world healthcare settings. Only one study has analyzed the impact of VBL on both bacterial inactivation and its influence on HAIs and hospital costs (43).

VBL’s potential economic benefits are significant. HAIs impose a substantial financial burden on healthcare systems, with an estimated average cost of $100,000 per HAI in the US (87). Studies suggest potential savings of millions due to HAI prevention with VBL (43). Murrell et al. consider a savings of about $900,000 due to the potential avoidance of 9 infections in their study. If personal liabilities, such as lost productivity, are considered, a single HAI would cost about $389,307–$474,004, with a savings in its prevention of about $3,500,000. The cost of HAIs is calculated based on the expenses needed for treatment, for hospitalization costs, whose time increases because of the infectious disease, and for the expenses to be invested in health care personnel and personal protective equipment needed to isolate the patient (43). The multidisciplinary teams involved in monitoring HAIs participate in audits following the occurrence of the event, and this results in a subtraction of time that could be channeled into other activities and thus economic loss. Staff illness in turn represents important economic relief, as does the increased mortality associated with HAIs (88). The economic benefits are represented not only by the reduction in HAIs but also using this specific technology, which has been shown to be economical and eco-friendly (27, 80).

VBL technology requires only one initial purchase and any low maintenance expenses but unlike chemical disinfectants does not require constant purchase and frequent procurement and storage (79, 89, 90). Finally, there is no need for the expense of hiring specialized technical staff or operators assigned exclusively to the activity (27). Staff can be trained on proper VBL use, fostering awareness and potentially reducing reliance on chemical disinfectants, leading to further cost savings (27, 43).

Obviously, we do not assume a replacement of traditional sanitation methods with the exclusive use of VBL, but rather a concomitant use of the methods. Thus, in general, there would not be a direct reduction in expenses associated with the use of the technology, but rather an economic return secondary to the reduction in HAIs.

To assess the real economic impact of a violet-blue light lamp, however, it would have to be ascertained how much it costs to operate the system itself, and especially what is the magnitude of the electrical expenses required to keep it lit.

The environmental impact of VBL is also positive. Unlike chemical disinfectants, VBL generates no harmful waste and is less deteriorating on materials or instruments (89). This translates to reduced waste disposal costs and a longer lifespan for medical equipment (44, 45). Additionally, VBL eliminates the environmental risks associated with chemical waste disposal (20, 89, 91–93).

It has been clear for years now how the environment in which we live is a determinant of our health status (94). Recently this aspect has been further investigated and climate and environmental ecology have been identified as determinants of the spread of antibiotic resistance (95).

VBL, if it causes an alteration in the structural heterogeneity of materials used in health care instrumentation, does so over long periods of time or at high doses, and this is associated with increased instrument safety (44, 45). In fact, the cracks formed, and structural heterogeneity can increase biofouling, prevent proper cleaning of the instrument, and thus increase the infectious risk to the patient (44). Alteration of the hydrophilic properties of the instrument and from its wettability result in alterations in the ability of bacteria to adhere (44).

In addition, the use of VBL enables disinfection of air and water, and the latter aspect could prove extremely important in health care, such as for inactivation in the water supply of Legionella (78, 96).

The use of VBL in disinfection of wounds or burns could be instrumental in changing patient outcomes (39–41). Intraoperative application could be particularly useful in orthopedic surgeries with prosthesis placement, as this surgery is the one most associated with the development of HAI (43, 45).

Its applicability in occupied rooms allows for continuous disinfection without requiring room closures, optimizing workflow and patient experience (47–55, 58).

However, some considerations regarding VBL use exist. In operating rooms, particularly during laparoscopic or robotic surgeries where procedures are performed in low-light environments, the use of light sources as disinfection tools must be evaluated and calibrated (43). Additionally, the potential effects of VBL on circadian rhythm, mood, and patient sensitivity, particularly for those with mental health conditions, require further study (97, 98). LED technology’s ability to mix colors offers promise for mitigating these concerns (43, 48, 86).

Among the limitations of the study, we must note the small number of research conducted on the efficacy of VBL in health care settings. In fact, only 6 studies have investigated the efficacy of violet-blue light in hospital/ambulatory settings. This may likely be related to the lack of knowledge of the mechanisms of action of VBL wavelength and the optimal timing for environmental disinfection. Knowledge of these parameters would allow the application of VBL technology *in vivo* to be optimized so that standard protocols can be drafted. However, since the Covid-19 pandemic, there has been a growing interest in this area, and it is likely that more and more data will be found in the literature in the coming years (35, 74, 99–102). Consequently, the lack of data correlating VBL technology and associated economic benefits will also be overcome. Indeed, as mentioned earlier, the economic savings are not direct and immediate, as the technology is certainly expensive, as is its maintenance (103). However, it is now known how HAIs negatively affect the economic budget of a health care facility and the costs associated with them are extremely high (6). Therefore, it is necessary to demonstrate how the use of VBL affects the performance of HAIs and how this is reflected at the economic level. The importance of infection control in healthcare settings and optimization of economic resources for public health coupled with the growing interest in alternative and safe disinfection technologies may lead to further exploration in this regard in the coming years.

In conclusion, while more research is needed, VBL appears to be a promising tool for healthcare disinfection. The available data suggests VBL’s effectiveness against a broad spectrum of pathogens (30, 39–43). The potential cost savings, environmental benefits, and improved patient outcomes associated with VBL use make it a worthwhile area for further investigation.

## Data Availability

The datasets presented in this study can be found in online repositories. The names of the repository/repositories and accession number(s) can be found in the article/Supplementary material.
